# Understanding UK medical students' perspectives on a career in cardiothoracic surgery

**DOI:** 10.1016/j.xjon.2021.08.035

**Published:** 2021-09-02

**Authors:** Ariana Axiaq, Renier A.B. Visser, Manasi Shirke, Arwa Khashkhusha, Sara Zaidi, Raneesha Pillay, Christopher J. Goulden, Amer Harky, Career Research Collaboration, Hugo Labat, Khubbaib Hasan, Marco Shiu Tsun Leung, Makishaa Nanthakumar

**Affiliations:** a School of Medicine, Queen's University Belfast, Belfast, United Kingdom; b School of Medicine, University of Central Lancashire, Lancashire, United Kingdom; c School of Medicine, University of Liverpool, Liverpool, United Kingdom; d King's College London, London, United Kingdom; e Bart's and the London School of Medicine, London, United Kingdom; f Imperial College School of Medicine, London, United Kingdom; g Faculty of Health and Life Sciences, School of Medicine, University of Liverpool, Liverpool, United Kingdom; h Department of Cardiothoracic Surgery, Liverpool Heart and Chest Hospital, Liverpool, United Kingdom; i Liverpool Centre for Cardiovascular Science, University of Liverpool and Liverpool Heart and Chest Hospital, Liverpool, United Kingdom

**Keywords:** perception, interest, career, cardiothoracic surgery, medical students, CV, cardiovascular, PGY, postgraduate year

## Abstract

**Objectives:**

The UK postgraduate training program in cardiothoracic surgery is challenging and competitive, with trainees choosing the field for different reasons. This study aims to identify factors that influence medical students in pursuing a career within cardiothoracic surgery.

**Methods:**

A cross-sectional study was carried out in which a questionnaire was anonymously filled out by medical students across 17 medical schools in the United Kingdom. An online survey platform was used for survey distribution and analysis. A mixed-methods approach was employed to gather quantitative and qualitative data. Data collection consisted of a series of closed questions and 1 open-ended question. The questions focused on the attitudes toward, knowledge of, and exposure to cardiothoracic surgery.

**Results:**

The survey yielded 265 responses. Interest in cardiothoracic surgery was seen in 45.3% of participants, with the leading factor for pursuing this career being lifestyle factors (50%), closely followed by the career opportunities (42.9%) and the aid of mentors (31%). Some discouraging factors were: Difficulty of learning material (37.7%), length of the training program (27.4%), competition in the field (26%), stress (24.3%), and lifestyle factors (22.1%).

**Conclusions:**

Whilst UK medical schools try to provide an introduction to specialties like cardiothoracic surgery, there remains a proportion of medical students who do not have access to opportunities needed to make a balanced career decision. Additionally, individual circumstances and aspirations tend to change from students' first year of study to their final year, which can alter their perceptions about the field.

## Graphical abstract

An overview of a survey study in a medical student population assessing their knowledge, interest, and exposure in cardiothoracic surgery and a career in the specialty. The main findings for the 3 primary study objectives have been presented together with a method summary and the final action points from the study. Values feature mean scores or percentage of total respondents.

**Figure:**
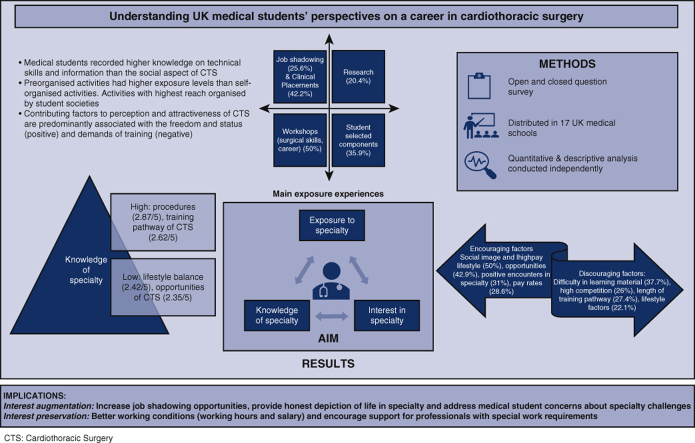

**Figure:**
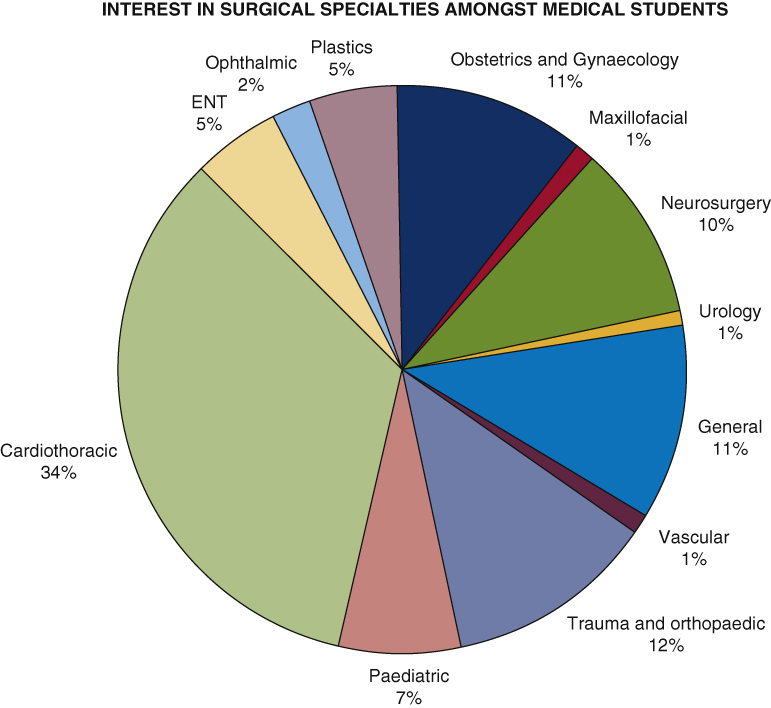
Pie chart depicting the proportion of students choosing each specialty.

**Central Message**

There are varied perceptions of cardiothoracic surgery careers amongst UK medical students. Research is needed to clarify these views and the reasons behind them for diverse and effective recruitment.

**Perspective**

Attracting enthusiastic individuals to the specialty is essential to the sustainability of cardiothoracic surgery. In this study, we aimed to understand the influences behind the perceptions of cardiothoracic surgery amongst UK medical students and identify strategies that could ameliorate specialty numbers, through the distribution of a multiuniversity survey targeting medical students of all years.

See Commentaries on pages 518, 520, and 522.

In today's age, the demand of cardiovascular disease on health care systems has increased globally and accounts for a staggering mortality rate of approximately 50% in developed countries.^1^ The subsequent disparity in supply and demand could lead to crucial shortages, potentially damaging the quality of patient care.^2^

It should be noted that cardiothoracic surgery is a common aspiration for many medical students at the start of their studies. A cohort study done in the United States, by Coyan and colleagues,^3^ found that 30% of first-year medical students were interested in cardiothoracic surgery. However, in all the other years the percentage interest decreased drastically and remained consistently below 15% (*P* = .02).

The decreasing interest amongst medical students has already influenced the number of applicants for cardiothoracic surgery training. In 2014, 2015, and 2016, the application rates for cardiothoracic surgery at early speciality trainee level in the United Kingdom was 100, whereas in 2017 there was a dramatic decrease to 80.^4^ Furthermore, a 2020 survey by Miller and colleagues^5^ found a significant difference in cardiothoracic surgery interest between genders, with men almost 2.5 times more interested than women. Factors such as exposure and representation were held accountable. Multiple training levels need to be completed to become a fully qualified cardiothoracic surgeon in the United Kingdom. Applicants typically can choose to specialize after completing their 2 foundation years (postgraduate year [PGY] 1-2). Early specialty training (PGY3-4) and higher specialty training (PGY5 onward) are required. Some applicants may opt to start run-through training where once-off recruitment is done in the beginning of the training program (PGY3 onward). Run-through training is a standard 8-year program where juniors in their first year are referred to as early specialty trainee year 1 and advanced trainees in their final year are referred to as higher specialty trainee year 8. Early exposure to cardiothoracic surgery in medical school years has been shown to have a significant influence on student interest. In 2019, a survey by Gasparini and colleagues^6^ showed a correlation between interest in cardiothoracic surgery and exposure, finding that medical students with curricular, clinical, or extracurricular exposure reported a higher interest and confidence in cardiothoracic surgery.

Through this study, we aimed to examine the factors that influence medical students' interest in pursuing a career in cardiothoracic surgery. We investigated factors including exposure opportunities, their interest compared with other specialties, and knowledge about different aspects of the cardiothoracic surgery specialty with the goal to better understanding what influences the decision to pursue cardiothoracic surgery as a career.

## Materials and Methods

Participants were asked to read a participant information sheet before starting the survey (Online Data Supplement 1). On the first page of the survey, a consent form was filled out to confirm they have read the participant information sheet. The information sheet confirmed they were aware that their responses would remain anonymous, no identifiable information would be collected, and their responses would not be used for anything else other than research purposes. This study was conducted with review number 10133 - University of Liverpool School of Medicine.

### Survey Production

The survey was produced using an online platform, Joint Information Systems Committee. To reduce the risk of duplicate responses, the survey platform only allowed 1 response from each device. The survey was created to gauge interest, and the cardiothoracic surgery exposure received from the current curriculum in respective medical schools. The questions featured in the survey were developed and unanimously agreed upon amongst all authors involved. Piloting of the survey was conducted amongst the authors before dissemination. The feedback obtained from the pilot was incorporated into the final survey upon distribution. The survey questions consisted of background and demographic characteristics, attitudes and determining factors of choosing a career in cardiothoracic surgery, and exposure to cardiothoracic surgery. There were 18 questions that consisted of multiple choice, rating, ranking, and short answer questions (Online Data Supplement 2).

### Survey Population

The survey was prepared and sent to 17 of 33 UK medical schools. The survey was not distributed to 16 medical schools also registered with the General Medical Council because collaborators from these schools could not be found. The survey was open to all medical students regardless of whether they had an interest in surgery or cardiothoracic surgery. The survey yielded 265 responses, which is notably low; there are an estimated 8730 medical school graduates overall in the United Kingdom as of 2019.

### Survey Distribution

Survey distribution began December 12, 2020, and responses were collected over a 3-week period. The survey was disseminated by means of E-mail messages, social media, and messaging services. Posters and quick-response codes were created to attract as many students as possible to take part in the survey (Online Data Supplement 3). However, the distribution methods used above meant it was not possible to determine how many students the survey was sent to; thus, a response rate could not be determined. Collaborators were appointed from each participating UK medical school to distribute the survey to their respective schools.

### Survey Analysis

Data were analyzed on the Joint Information Systems Committee platform itself based on the number of responses received and through cross-tabulation. The descriptive nature of the study resulted in the analysis being more qualitative than quantitative.

## Results

### Background

**Figure 1:**
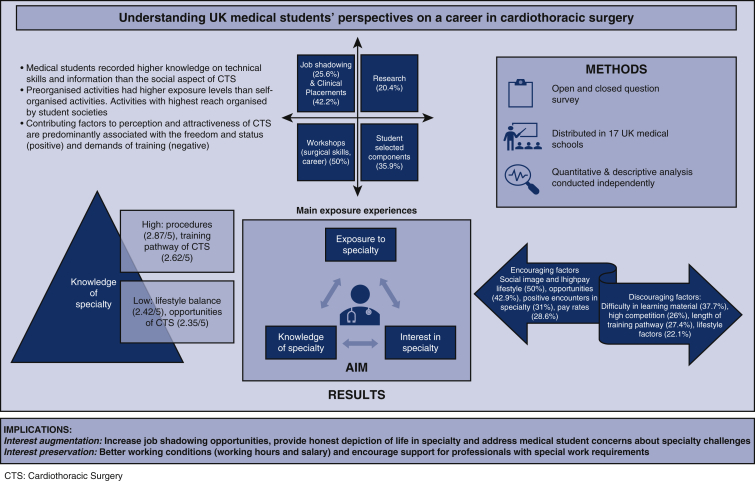
A summary of our study explaining the findings of survey results of 265 students among 17 medical schools in the United Kingdom (*UK*). We aimed to understand the knowledge and interest in cardiothoracic surgery speciality among the undergraduates. The study has identified several factors that can possibly be utilized for recruiting future cardiothoracic surgery (*CTS*) trainees.

Our survey received 265 responses from students across 17 medical schools within the United Kingdom. Of these respondents, the majority identified as “Female” (58.9%) with only 40.8% identifying as “Male.” Among respondents, 66.1% were in their clinical years of medical school (PGY3-5) and 10.9% of the total participants were presently studying an intercalated degree (Figure 1). An intercalated degree is an extra year of study often taken by medical students to study a field of one's choice at greater depth.

### Attitudes and Determining Factors

**Figure 2:**
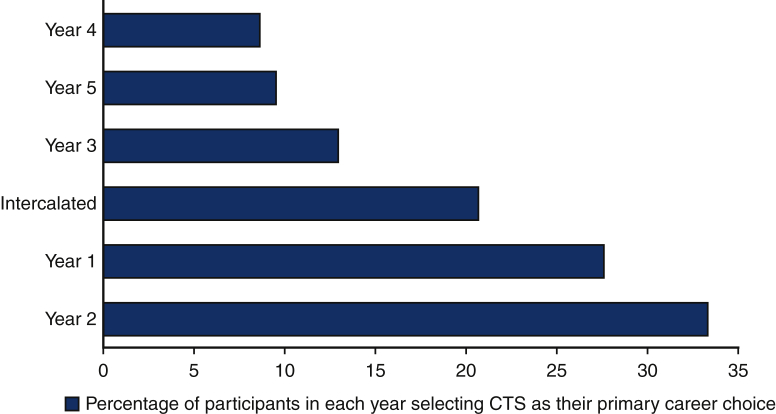
A graph showing the percentage of participants with a primary interest in cardiothoracic surgery against the year group they are in. *CTS*, Cardiothoracic surgery.

Out of the 265 medical students from PGY1 through PGY5 who took the survey, 76.6% (203) knew in which speciality they would like to pursue a career. Among the survey takers, 45.3% (120) were keen to pursue a career in surgery. Additionally, 93 (35.1%) were interested in medicine and a few were keen on a career in primary care. Out of the 120 students who were interested in surgery, there was a general preponderance toward cardiothoracic surgery (Figure 2). On further questioning, the main reason for an interest in cardiothoracic surgery as a career varied between individuals, ranging from lifestyle factors (the social image and lifestyle that the salary as a cardiothoracic surgeon could provide) (50%), opportunities in specialty (42.9%), mentors and previous encounters (31%), pay rates (28.6%), decision-making process (27.5%), and adequate previous exposure (32.1%).

**Figure 3:**
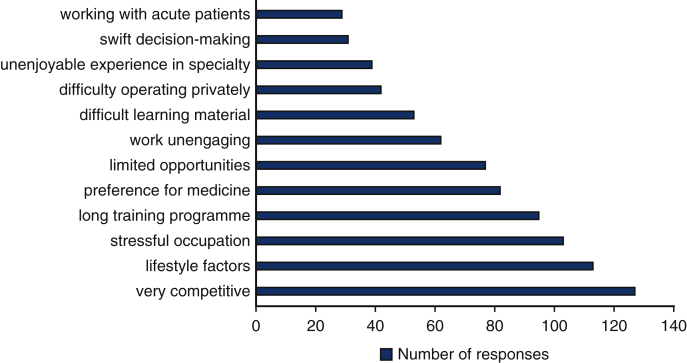
Frequency of options chosen as to why the students are not interested in becoming cardiothoracic surgeons.

A similar system of grading was implemented to gain an insight into the reasons why students were not interested in pursuing a career in cardiothoracic surgery. The reasons ranged from difficulty of learning material (37.7%), length of training program (27.4%), competition (26%), stress (24.3%), and lifestyle factors (poor work–life balance and long working hours) (22.1%) (Figure 3). Moreover, 156 out of the total number of 265 students also preferred medicine over surgery. By contrast, the majority of students (63.2%) were open to considering a career in cardiothoracic surgery in the future despite its potential perceived limitations. A few people were concerned about cardiothoracic surgery becoming a dying profession. Other participants have had experiences of the cardiothoracic surgery staff and environment as being hostile.

### Knowledge of the Specialty

Additional inquiries were made on what factors influence student understanding of the working life of a cardiothoracic surgeon. Medical students felt most informed on the procedures typically done in cardiothoracic surgery (a mean score of 2.87 out of 5) followed by “structure of the training pathway” (2.62 out of 5) and the skills required to be a cardiothoracic surgeon (2.53 out of 5). Slightly lower mean scores were obtained for the working hours of a typical cardiothoracic surgeon (2.46 out of 5) as well as their work–life balance (2.42 out of 5); with respondents being least confident about their knowledge on opportunities available in cardiothoracic surgery.

### Medical School Exposure to Cardiothoracic Surgery

From the 265 respondents, 20.4% answered that they have done or are currently doing a degree in cardiovascular (CV) science, or a degree that involved CV research. It was also revealed that 53.1% of the participants were exposed to cardiothoracic surgery before medical school, during medical school, or both. The 2 most reported experiences were through clinical placements (42.2%) or self-organized job shadowing (25.6%). Surprisingly, 22.2% of participants had no exposure to cardiothoracic surgery.

More than half of the respondents were not aware of career support services offered by their medical school and only 20.3% confirmed that these were available at their medical school. It was found that cardiothoracic surgery events in medical school were mostly student-led, consisting of society events (81.3%) and surgical skills workshops (50%). University-organized events were less common but still prevalent, with 40.6% having special or student selected components with 35.9% having the option of a cardiothoracic-related clinical placement to explore their interest. Activities such as mentorships and CV sessions were the least reported. 23.7% of respondents reported that the above-mentioned events increased their interest in the specialty. Some respondents were discouraged by the competitive and high-pressure aspect of the specialty portrayed by the above events.

## Discussion

### Summary

Findings from this study have revealed trends that contrast and add to previous reports. The patterns observed provide an insight into the current perceptions of a career in cardiothoracic surgery amongst UK medical students, with an aim to inform future planning and interventions to improve current attitudes and awareness.

### Attitudes and Determining Factors

Of the respondents wanting to pursue a surgical specialty, 34% stated that cardiothoracic surgery is the specialty in which they had the most interest. This may be explained by the survey being circulated by cardiothoracic surgery university societies, with students interested in pursuing a career in cardiothoracic surgery more likely to engage in a research survey with a cardiothoracic surgery focus. Our findings mirror those of Gasparini and colleagues^6^ and Preece and colleagues,^7^ with exposure playing a crucial role in expanding interest in cardiothoracic surgery. The types of exposure linked with the highest interest in pursuing cardiothoracic surgery were: “Research” (34.8%), “Surgical teaching during cardiothoracic anatomy” (21.2%), and “Job shadowing” (19.6%). This builds on the findings of Berger and colleagues^8^ who also found involvement in research to be a factor that significantly increases students interests in pursuing a career in a surgical specialty.

However, it must be considered that students with a preexisting interest in cardiothoracic surgery are more likely to seek out opportunities to perform cardiothoracic surgery research. Figure 2 shows that interest in pursuing cardiothoracic surgery is high with preclinical students (PGY1-2), then decreasing dramatically throughout the clinical year students (PGY3-5). This trend has also been seen in studies done by Preece and colleagues^7^ and Coyan and colleagues,^3^ with the former citing a lack of exposure as the reason for this. However, the decrease in interest could be for the converse reasons, with gaining cardiothoracic surgery experience exposing students to negative aspects of the career of which they were unaware.

**Figure 4:**
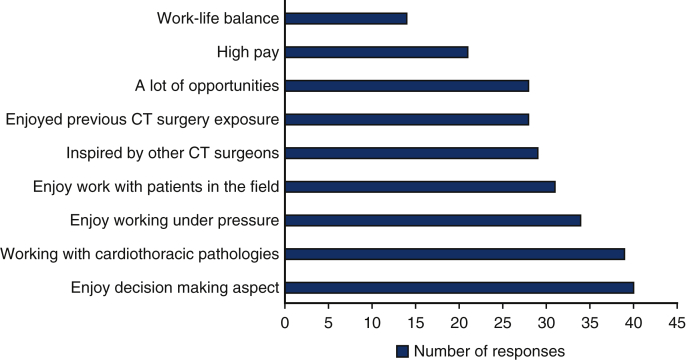
Graph depicting the frequency of options chosen as to why medical students are interested in pursuing a career in cardiothoracic (*CT*) surgery.

Mentorship and research opportunities positively correlate to student interest and participation in a surgical career. It allows the development of positive relationships with surgical mentors to gain a balanced understanding of the realities of a career in a related speciality.^9^ A Nigerian study noted significant factors that affected the interest of medical students in cardiothoracic surgery (Figure 4): the majority of the students described adequate exposure, mentorship, lifestyle factors (social image and lifestyle that a high salary could provide), and pay to be deciding factors for a career in the field,^10^ which run parallel to the findings in our study. Moreover, a US-based study found that students are drawn to the specialty due to the nature of skills required in addition to the intellectual nature of the clinical work. With surgical selection undergoing a dramatic change in the United Kingdom, successful selection in the competitive application process demands thorough preparation and keen interest and commitment beginning in medical school.^11^ These changes involve greater emphasis on experience in surgical cases, evidence of research, teaching, and leadership roles as well as completion of surgical courses.

As shown in Figure 3, cardiothoracic surgery being “Very competitive” was the most frequently chosen deterrent of a career in cardiothoracic surgery to the respondents. This is supported by cardiothoracic surgery having competition ratios of 9.92 for early specialty training applications and 5.86 for higher specialty training applications in 2020.^12^ “Long working hours” was the second most chosen factor as to why students do not want a cardiothoracic surgery career, which is echoed by Preece and colleagues,^7^ where it was the most common reason. Lifestyle factors or poor work–life balance is an unfortunate reality that many cardiothoracic surgeons face that discourages many of those who want to balance home and work life.^13^^,^^14^ The third most picked factor, “Stressful job,” also showed similar results to Preece and colleagues,^7^ where stress was cited as their second most common reason for not wanting to pursue a career in cardiothoracic surgery. It is evident that major deterrents to students wanting to be cardiothoracic surgeons are the systemic factors of the field. Increasing training posts for cardiothoracic surgery can address the above-mentioned factors because it will reduce the competitiveness and increase the future workforce of cardiothoracic surgeons, hopefully leading to a reduction in work pressure and an improved work–life balance for the surgeons.

Out of the 12 options provided as to why respondents do not want to pursue a career in cardiothoracic surgery, only 2 of the options did not have the majority of responses coming from students who had no cardiothoracic surgery exposure. This further supports that decreased exposure to cardiothoracic surgery in medical school leads to decreased interest in cardiothoracic surgery as a career. Thereby, if there is an increase in cardiothoracic surgery exposure incorporated into medical schools' curricula, this could lead to students being better informed of what being a cardiothoracic surgeon entails, which could increase interest.

A trend was observed in students excluding cardiothoracic surgery as a future job option; as medical students progress through medical school, interest in pursuing cardiothoracic surgery decreases accordingly. A possible explanation for this could be that as medical students go through their training, they are exposed to a larger range of specialties and have a better understanding of their career preferences. Among individuals declaring declaring that they have no future interest in pursuing cardiothoracic surgery, 37.5% also reported having no prior exposure to the specialty. This could be due to preconceived prejudices about the field, which could be challenged by offering more taster sessions and greater incorporation of cardiothoracic anatomy and surgery in the medical curriculum. A study by Haubert and colleagues^15^ assessed the influence on first-year US medical students of a surgical anatomy curriculum, compared with a standard anatomy module. Despite the academic improvement from the intervention being nonsignificant, students grew more appreciative of surgeons and more confident in anatomy.^15^ Despite the differences in the training programs in the United States, United Kingdom, and the rest of the world, the willingness of medical students to enter a surgical program is decreasing. Tambyraja and colleagues^16^ chose to evaluate the choice of teaching hospitals and overall interest in surgery. There was a general trend for students who had been attached to teaching hospitals to be less willing to consider surgery than those undertaking their placements in nonteaching hospitals—reasons for this were not explored.

In our study, 59.0% of respondents that eliminated cardiothoracic surgery as a choice also answered that they preferred medicine over surgery; it could be that disinterest in cardiothoracic surgery is due to it being a surgical specialty rather than due to the specialty itself. A surgical career is both rewarding and challenging, with significant hurdles to professional and personal life. Although job supply and demand vary worldwide, it is safe to assume that the general CV risk factors in the population are increasing, leading to an ever rising demand for more cardiothoracic surgeon specialists. Cardiothoracic surgery is already a niche specialty that requires extensive training in surgical management. A decreasing workforce supply with rising work pressures may lead to surgeon burnout, increased workload, and inevitably poorer standards of care and increased patient mortality.^2^

Participants who want to perform surgery but not cardiothoracic surgery specifically reported that this was due to a lack of exposure, further emphasizing a need for it to be improved. Some participants reported that they have experienced or have perceptions of cardiothoracic surgery being a hostile (ie, incidents of bullying) working environment. If this is the case, then systemic change is needed to deal with this because not only is it not healthy for staff to work in such an environment, but this might also deter potential future cardiothoracic surgery surgeons from pursuing it as a career. If not, cardiothoracic surgery having this reputation alone is going to have a negative influence on interest that could be remedied by students having more experience within the field, allowing them to assess the work environment for themselves.^14^

### Knowledge of the Specialty

Whilst higher mean estimates were obtained for medical students' knowledge of the skills possessed by a cardiothoracic surgeon; the training pathway, and the procedures part of a surgeon's daily routine; there was a general lack of awareness of the working hours and lifestyle of a cardiothoracic surgeon. This emphasizes that information about the technical side of the specialty is more readily available and abundant than information on the social and interpersonal aspects of the career. Building a coherent foundation of surgical knowledge is essential for medical teaching but for true engagement, a more honest portrayal of the lifestyle benefits, challenges within the field, and the support available should be provided. An example would be more open communication during clinical placements and encouraging questions that encompass the job experience rather than exclusively surgical knowledge, hopefully leading to establishing more balanced expectations.

### Medical School Exposure to Cardiothoracic Surgery

From our results, 20.3% of respondents have done, or are currently doing, a degree pertaining to cardiovascular physiology and science. However, from this group, the number of individuals preferring surgery equal those that prefer medicine (35.2%); with 27.8% of this group stating that they did not consider cardiothoracic surgery as a potential career. Studies have shown that intercalated degree students are more likely to follow academic careers,^17^ but whether or not this would be in academic surgery has yet to be determined.

**Figure 5:**
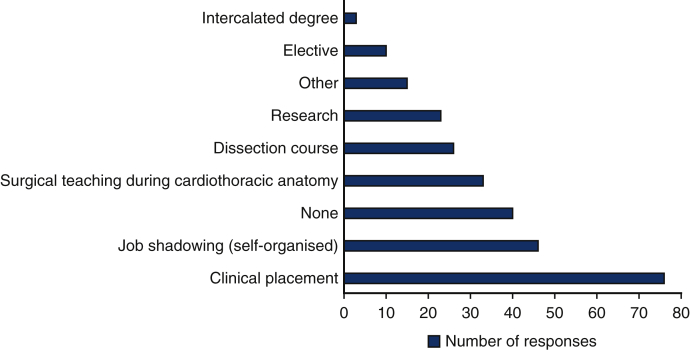
A graph showing undergraduate exposure to cardiothoracic surgery. The number of responses for each type of exposure is shown against each type of exposure.

More than half of those who reported an increase in interest from cardiothoracic surgery-related awareness activities, also took an initiative to organize job shadowing opportunities and a high proportion got involved in research. The highest reported exposure experience were clinical placements (42.2%) and self-organized placements (25.6%) followed by surgical teaching during cardiothoracic anatomy (18.3%) (Figure 5). Whilst medical schools have traditionally provided cardiothoracic surgery experience, new routes to gain exposure have been created by external organizations such as the Society for Cardiothoracic Surgery and the Royal College of Surgeons.^18^ This may lead the way for more modern approaches to collaborative medical teaching and shared responsibility in the exposure of cardiothoracic surgery.

We found that 58.9% of students were not aware of the guidance and training provided by their medical schools for a career in cardiothoracic surgery. Only 20% were aware of the opportunities provided by their medical school. Students' interest in the field increased due to seminars, conferences, mentoring, career advice, and placements. Experience in clinical placements has been shown to be influential in future career decision making. Clinical exposure in seemingly niche specialties like cardiothoracic surgery is essential as it exposes students to typical diagnostic and intellectual challenges; it also allows them the opportunity to interact with potential role models.^18^ However, most people did not find their interest in the field to fluctuate despite the resources offered by medical schools. Early exposure to courses focused on the field has been shown to improve interest in the specialty.^18^ It is interesting to note that a good proportion of students gained exposure to the field by means of medical society events (81.3%), student selected components (40.6%), placements (35.9%), research (20.3%), and mentoring opportunities (15.6%) during their time in medical school. However, most of the students' interest in the field remained unchanged (71%) despite additional aid from the university as well as student-led bodies.

### Limitations

With the aim to eliminate bias, data collectors were instructed to distribute the survey on social media that has a medical student only or medical student rich audience such as medicine year group pages. However, certain selection bias was inevitable, resulting in several limitations in this study. Firstly, nonmedical students could have answered the survey and students with an interest in cardiothoracic surgery might have been keener to answer the survey. Given the qualitative nature of data collected, responses might have been subjective due to the respondents' own understanding of the questions. Response rate is <20%, mainly owing to the limited reach of social media platforms used. This may have resulted in a sample unrepresentative of the medical student population, in terms of gender, background, and geography. Furthermore, participants may have differing opinions, different levels of exposure depending on their medical school, and subsequently different levels of knowledge of cardiothoracic surgery. These limitations due to the nonstandardized nature of medical education within the United Kingdom may therefore not represent all medical students. Therefore, further studies with a larger response rate from all medical schools in the United Kingdom need to be conducted to come to a more accurate and representative conclusion. Additionally, the increase in endovascular procedures and subsequent interest in interventional cardiology could have been explored as an influential factor.

### Future Research and Improving Cardiothoracic Surgery Opportunities

For our study to make a positive impact on the exposure opportunities in cardiothoracic surgery for medical students, it is crucial to first widen the number of participants. Although 265 is a substantial figure, obtaining responses from an even larger cohort of medical students would be most favorable and address some of the inevitable limitations of the survey format.

Our survey found that more than half of medical students were not aware of guidance and training opportunities for a career in cardiothoracic surgery, tackling this area could lead to an improvement in medical student perception of cardiothoracic surgery.^7^^,^^18^ This can be achieved through shadowing surgeons and requiring medical schools to include a cardiothoracic surgery rotation during clinical years. An engaging experience, with an active role on the surgical ward and in theatre could provide a significant influence on perceptions.^6^, ^7^, ^8^^,^^10^ In addition, other campaigning strategies such as frequent live webinars that involving a range of surgeons speaking about what their work–life balance, would help address many medical student concerns. Other incentives such as increased wage and a reduction in nonsocial working hours should also be negotiated and advertised. Furthermore, an increase in abstract submission and poster presentation competition would also support increasing cardiothoracic surgery exposure.^6^, ^7^, ^8^^,^^10^^,^^18^

A further step could be to address students' concerns and build an environment where students feel comfortable, confident and empowered to seek opportunities that they may not have known existed and even create them to gain exposure to cardiothoracic surgery as a career. This could be in the form of integrating cardiovascular medicine societies from a range of UK medical schools by running interuniversity conferences (such as the National Undergraduate Cardiovascular Conference, delivered by King's College London), setting up workshops (collaborating with the Association of Surgeons in Training) and research and mentorship schemes (increase intake of students for abstract submissions with the Society for Cardiothoracic Surgery).^6^, ^7^, ^8^^,^^10^^,^^18^ These could also be implemented worldwide using respective nations' equivalent societies and organizations. This nationwide access would address many of these concerns, and the feedback collected from sessions could also be used to improve services for future students and monitor how their perceptions of cardiothoracic surgery has changed after access to these events and services.

## Conclusions

The potential of a future shortage of cardiothoracic surgeons has led to a spurt of surveys and studies to find and combat the underlying cause. This study has shown that medical students are less likely to want a career in cardiothoracic surgery as they progress through medical school. Action must be taken to initiate and maintain interest in cardiothoracic surgery throughout student doctor training. Factors influencing student opinion appear to be centered on a negative perception due to a lack of exposure, mentorship, and extracurricular opportunities. The result has been a decline in applicants to cardiothoracic surgery and a growing concern over the future of the workforce. There needs to be prompt action by medical schools and national organizations to help engage and highlight the attraction of a career in cardiothoracic surgery.

### Conflict of Interest Statement

The authors reported no conflicts of interest.

The *Journal* policy requires editors and reviewers to disclose conflicts of interest and to decline handling or reviewing manuscripts for which they may have a conflict of interest. The editors and reviewers of this article have no conflicts of interest.
