# Supplementary material for: Understanding UK medical students' perspectives on a career in cardiothoracic surgery
Source: JTCVS Open. 2021 Sep 2;8:509–17. doi: 10.1016/j.xjon.2021.08.035 (PMC9390747; doi:10.1016/j.xjon.2021.08.035)
Supplement: Online Data Supplement 1 [file mmc1.docx]

You are being invited to participate in a research study titled:

**Career choice among medical students: are we losing future cardiac surgeons?**

**AIM OF THE STUDY**

This 10 minute survey is designed to evaluate the perceptions of cardiothoracic surgery in particular cardiac surgery as a career. Data collected from this questionnaire will be formulated into results. Outcomes of the study will be submitted for publication in a peer reviewed journal with the purpose of providing a more realistic perception of cardiac surgery and improving recruitment for cardiothoracic and cardiac surgery training programmes.

**WHY HAVE I BEEN INVITED TO PARTICIPATE IN THIS STUDY?**This survey focuses on perceptions of all medical students attending medical schools in the United Kingdom.

**PARTICIPATION IN THE SURVEY**Participation is on a voluntary basis and one can choose to opt out before filling in the survey or any time before submitting

After reading this information sheet, you can consent to taking part in this study and start the questionnaire.

Whilst participation does not present with any risks or personal benefit, this study is aimed at investigating current student engagement in cardiac surgery and perception of medical students about cardiac surgery as a career. Outcomes from this study may guide further action to improve medical student resources about careers in cardiac surgery.

**IS MY CONTRIBUTION IN THE SURVEY ANONYMOUS?**

Given that participants read the information sheet and consented to taking part, participants will be allowed to start the survey. Participation is completely voluntary, and responses will not be linked to the respondent. To ensure anonymity, data collected will be coded hence survey responses may not be identifiable.

**HOW WILL RESULTS BE USED FROM THIS STUDY?**

Data collected from this survey will be anonymised prior to analysis. Subsequent results will also be anonymised including any presence of qualitative data such as quotations. Outcomes from the study may be published in a peer-reviewed journal and possibly presented in a conference. Anonymity will be maintained.

**STUDY ETHICAL REVIEW**

This project has been reviewed and approved by the University of Liverpool Ethics Committee. If you have any questions or concerns, please contact the chief investigator of this study, Dr Amer Harky via email: [aaharky@liverpool.ac.uk](mailto:aaharky@liverpool.ac.uk) / [aaharky@gmail.com](mailto:aaharky@gmail.com) .
