# Supplementary material for: Understanding UK medical students' perspectives on a career in cardiothoracic surgery
Source: JTCVS Open. 2021 Sep 2;8:509–17. doi: 10.1016/j.xjon.2021.08.035 (PMC9390747; doi:10.1016/j.xjon.2021.08.035)
Supplement: Online Data Supplement 2 [file mmc2.pdf]

Career choice among medical students: are we losing future cardiothoracic surgeons?

Page 1: You have been invited to participate in a survey research study titled: Career choice among medical students: are we losing future cardiothoracic surgeons?

In order to consent to participation in the survey, you need to confirm that:

1. You have read and understood the participant information sheet.

2. You are aware of your ability to ask any questions about the study provided you have any concerns.

3. You understand that data collected from responses will be kept confidential and anonymised so that individual respondents cannot be identified.

4. You are aware that information gathered will be managed, stored and discarded securely. You understand that individuals analysing the data will have access to responses and that data collected may be used for further research purposes.

5. You are aware of participation in this study being entirely voluntarily and that you can withdraw from this study any time before submitting your responses.

6. You are over the age of 18 years.

If you agree with all six statements, you may proceed to the survey.

Page 2: Background

1. Which medical school are you currently attending? ✱ *Required*

2. Which year of medical school are you at (2020/21)? ✱ *Required*

- Year 1
- Year 2
- Year 3
- Year 4
- Year 5
- Intercalated year

3. Do you have a previous degree/ are you currently doing a degree in cardiovascular science or a BSc degree with cardiovascular research? ✱ *Required*

- Yes
- No

4. Which gender do you identify with? ✱ *Required*

- Male
- Female
- Other
- I prefer not to say

Page 3: Exposure to cardiothoracic surgery

5. Have you been exposed to cardiothoracic surgery before or during medical school? *
Required

- Yes, before medical school
- Yes, during medical school
- Yes, both before and during medical school
- No

6. If you replied ‘yes’ to Q5, how have you been exposed to cardiothoracic surgery?

- Clinical placement
- Elective
- Research
- Intercalated degree
- Job shadowing (self-organised)
- Surgical teaching during cardiothoracic anatomy
- Dissection course
- Other
- None

If you selected Other, please specify:

Page 4: Knowledge about cardiothoracic surgery as a career

7. On a scale from 1 to 5 how do you rate your understanding of cardiothoracic surgery in terms of the following (1 being very low to 5 being very high)? ✱ *Required*

Please don't select more than 1 answer(s) per row.

Please select at least 1 answer(s).

	1 (very low)	2 (low)	3 (moderate)	4 (high)	5 (very high)
Length of training pathway	☐	☐	☐	☐	☐
Working hours of a cardiothoracic surgeon	☐	☐	☐	☐	☐
Skills required to become a cardiothoracic surgeon	☐	☐	☐	☐	☐
Opportunities in cardiothoracic surgery	☐	☐	☐	☐	☐
Procedures performed by a cardiothoracic surgeon	☐	☐	☐	☐	☐
Work-life balance of a cardiothoracic surgeon	☐	☐	☐	☐	☐

8. Does your medical school provide guidance and training for a career in cardiothoracic surgery? ✱ *Required*

- Yes
- No
- I am not aware

9. If you replied ‘yes’ to Q8, how does your medical school encourage and support interest in cardiothoracic surgery?

- CV stations/ portfolio building sessions
- Medical society events
- Surgical skill workshop
- SSC with focus on cardiothoracic surgery
- Placements in cardiothoracic surgery
- Research opportunities
- Mentoring for keen students
- Career advisory services
- Career seminars
- Other

If you selected Other, please specify:

10. How did the activities in Q9 influence your interest in pursuing cardiothoracic surgery as a career?

- Increased my interest
- Decreased my interest
- Did not affect my interest

11. If the activities in Q9 decreased or increased your interest in pursuing cardiothoracic surgery as a career, explain why.

Page 5: Attitudes about cardiothoracic surgery as a career

12. Do you have an idea of which specialty you would like to join in the future? ✱ *Required*

- Yes
- No

13. Would you prefer joining a medical specialty or a surgical specialty? ✱ *Required*

- Medicine
- Surgery
- I do not know
- Other

If you selected Other, please specify:

14. **If you answered surgery to Q13**, which surgical specialty would you most consider choosing?

15. **If you chose cardiothoracic surgery in Q14**, what are the 5 main reasons for this? (Only select 1-5 for the 5 top reasons – 1 being the top reason) (optional)

Please don't select more than 1 answer(s) per row.

Please don't select more than 5 answer(s).

	1	2	3	4	5
I enjoyed previous exposure experiences in cardiothoracic surgery	☐	☐	☐	☐	☐
Enjoy decision making aspect of the job	☐	☐	☐	☐	☐
Enjoy working under pressure	☐	☐	☐	☐	☐
High pay	☐	☐	☐	☐	☐
Lifestyle factors	☐	☐	☐	☐	☐
Enjoy working with cardiothoracic pathologies	☐	☐	☐	☐	☐
A lot of opportunities	☐	☐	☐	☐	☐
I enjoy working with patients usually encountered in cardiothoracic surgery	☐	☐	☐	☐	☐
Inspired by previous encounters with cardiothoracic surgeons	☐	☐	☐	☐	☐

Page 6: Attitudes about cardiothoracic surgery as a career

16. If you did not choose cardiothoracic surgery in Q14, what are the 5 main reasons for this? (Only tick 1-5 for the top 5 reasons – 1 being the most important reason) (Optional)

Please don't select more than 1 answer(s) per row.

Please don't select more than 5 answer(s).

	1	2	3	4	5
Learning material is difficult	☐	☐	☐	☐	☐
Did not enjoy my placement/elective in cardiothoracic surgery	☐	☐	☐	☐	☐
Long training programme	☐	☐	☐	☐	☐
Very competitive	☐	☐	☐	☐	☐
Limited opportunities	☐	☐	☐	☐	☐
Stressful job	☐	☐	☐	☐	☐
Do not enjoy making important decisions on the spot	☐	☐	☐	☐	☐
Do not find the work the working engaging/interesting	☐	☐	☐	☐	☐
Lifestyle factors (working hours etc.)	☐	☐	☐	☐	☐
Hard to do private practice work	☐	☐	☐	☐	☐
Prefer working with non-acute patients	☐	☐	☐	☐	☐

I would prefer medicine over surgery	☐	☐	☐	☐	☐

17. If you did not choose cardiothoracic surgery in Q14, would you still consider it an option in the future?

- ○ Yes
- ○ No

18. If you are interested in surgery but did not choose cardiothoracic surgery, what was the main reason(s) for this?

Page 7: Final page

Thank you for taking the time to answer our survey.

If you have any questions, please contact aaharky@liverpool.ac.uk

---

Key for selection options

1 - 1. Which medical school are you currently attending?

Anglia Ruskin University
Aston University
Barts and the London (QMUL)
Brighton and Sussex Medical School
Cardiff University
Edge Hill University Medical School
Hull York Medical School
Imperial College London
Keele University School of Medicine
Kent and Medway Medical School
King's College London
Lancaster University
London School of Hygiene & Tropical Medicine
Newcastle University School
Norwich Medical School
Plymouth University Peninsula Schools of Medicine and Dentistry
Queen's University Belfast
St George's, University of London
Swansea University
The University of Edinburgh
University College London
University of Aberdeen
University of Birmingham
University of Bristol
University of Buckingham
University of Cambridge
University of Central Lancashire

University of Dundee
University of Exeter
University of Glasgow School of Medicine
University of Leeds
University of Leicester
University of Liverpool
University of Manchester
University of Nottingham - Lincoln Medical School
University of Nottingham School of Medicine
University of Oxford
University of Sheffield
University of Southampton
University of St Andrews
University of Sunderland
University of Warwick
University of East Anglia

14 - 14. If you answered surgery to Q13, which surgical specialty would you most consider choosing?

Obstetrics and Gynaecology
Neurosurgery
Paediatric surgery
General surgery
Trauma and orthopaedic surgery
Cardiothoracic surgery
Plastic surgery
Vascular surgery
Urology surgery
ENT surgery
Maxillofacial surgery
Ophthalmic surgery
