# Supplementary material for: Understanding UK medical students' perspectives on a career in cardiothoracic surgery
Source: JTCVS Open. 2021 Sep 2;8:509–17. doi: 10.1016/j.xjon.2021.08.035 (PMC9390747; doi:10.1016/j.xjon.2021.08.035)

**CT Surgery Survey Advertisements**

- Hello! Would you mind filling out our survey?

It is to research medical students’ opinions as to whether or not you would like to become a Cardiothoracic Surgeon and your reasonings behind your decision.

We would really appreciate it if you could just spare a few minutes and follow the link below to complete our survey. Thank you!

- Calling all medical students!!!!

We would like to hear from you about your opinion as to whether or not Cardiothoracic surgery is one of your potential career prospects and why.

Follow the link below and let us know what you think?

- You!!! Yes, I'm talking to you! We need your help!

Can you fill in our survey giving us your opinion on Cardiothoracic surgery as a potential career prospect? Then follow the link below!

- Are you considering becoming a Cardiothoracic surgeon? Are you definitely NOT? Or do you fall in between these two groups?

If so, please help us by filling in the survey below so that we can gather your opinions on how you view Cardiothoracic surgeon as a career.

- Hi! We have a survey down below that will only take a few minutes to fill in.

Your opinion will help us determine what attracts and steers people away from a career in Cardiothoracic surgery. So please help us find ways that open up this career path to many more people!


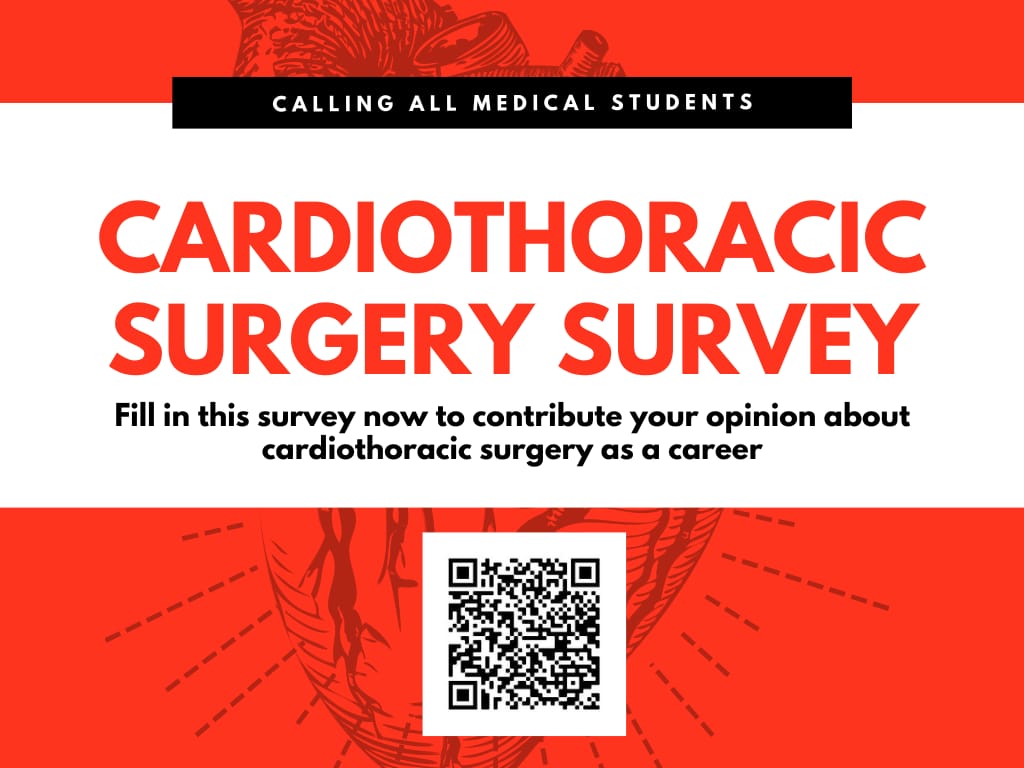

Supplement: Online Data Supplement 3 [file mmc3.docx]
